# CCN3 Signaling Is Differently Regulated in Placental Diseases Preeclampsia and Abnormally Invasive Placenta

**DOI:** 10.3389/fendo.2020.597549

**Published:** 2020-11-16

**Authors:** Liyan Duan, Manuela Schimmelmann, Yuqing Wu, Beatrix Reisch, Marijke Faas, Rainer Kimmig, Elke Winterhager, Angela Köninger, Alexandra Gellhaus

**Affiliations:** ^1^Department of Gynecology and Obstetrics, University of Duisburg-Essen, Essen, Germany; ^2^Institute of Molecular Biology, University of Duisburg-Essen, Essen, Germany; ^3^Department of Pathology and Medical Biology, University Medical Centre Groningen, Groningen, Netherlands; ^4^Electron Microscopy Unit (EMU)/Imaging Center Essen (IMCES), University of Duisburg-Essen, Essen, Germany

**Keywords:** CCN3, trophoblast, senescence, invasion, preeclampsia, abnormally invasive placenta

## Abstract

**Objectives:**

An adequate development of the placenta includes trophoblast differentiation with the processes of trophoblast migration, invasion, cellular senescence and apoptosis which are all crucial to establishing a successful pregnancy. Altered placental development and function lead to placental diseases such as preeclampsia (PE) which is mainly characterized by insufficient trophoblast invasion and abnormally invasive placenta (AIP) disorders (*Placenta accreta*, *increta*, or *percreta)* which are characterized by excessive trophoblast invasion. Both of them will cause maternal and fetal morbidity/mortality. However, the etiology of these diseases is still unclear. Our previous study has shown that the matricellular protein *nephroblastoma overexpressed* (NOV, CCN3) induces G0/G1 cell cycle arrest, drives trophoblast cells into senescence and activates FAK and Akt kinases resulting in reduced cell proliferation and enhanced migration capability of the human trophoblast cell line SGHPL-5. The present study focuses on whether CCN3 can alter cell cycle-regulated pathways associated with trophoblast senescence and invasion activity in pathological versus gestational age-matched control placentas.

**Methods:**

Cell cycle regulator proteins were investigated by immunoblotting and qPCR. For localization of CCN3, p16, p21, and Cyclin D1 proteins, co-immunohistochemistry was performed.

**Results:**

In early-onset PE placentas, CCN3 was expressed at a significantly lower level compared to gestational age-matched controls. The decrease of CCN3 level is associated with an increase in p53, Cyclin E1 and pRb protein expression, whereas the level of cleaved Notch-1, p21, Cyclin D1, pFAK, pAKT, and pmTOR protein decreased. In term AIP placentas, the expression of CCN3 was significantly increased compared to matched term controls. This increase was correlated to an increase in p53, p16, p21, Cyclin D1, cleaved Notch-1, pFAK, pAkt, and pmTOR whereas pRb was significantly decreased. However, in late PE and early AIP placentas, no significant differences in CCN3, p16, p21, Cyclin D1, p53, and cleaved Notch-1 expression were found when matched to appropriate controls.

**Conclusions:**

CCN3 expression levels are correlated to markers of cell cycle arrest oppositely in PE and AIP by activating the FAK/AKT pathway in AIP or down-regulating in PE. This may be one mechanism to explain the different pathological features of placental diseases, PE and AIP.

## Introduction

The cytotrophoblast (CTB) cells come from the outermost layer of the blastocyst. They are highly proliferative in nature and can give rise to two phenotypes: On one hand, they differentiate into the terminally differentiated multinucleated syncytiotrophoblast (STB) through cell fusion (1). The STB secretes hormones needed for pregnancy maintenance and forms a protecting barrier that regulates the transplacental transport such as the delivery of nutrients and gas to the fetus and the disposal of waste. On the other hand, the CTB cells detach from placental villi and differentiate into interstitial trophoblasts (iEVTs) or endovascular trophoblasts (eEVTs) (1). They take over another most important mission as invading into the maternal decidua to open up maternal spiral arteries to provide sufficient blood flow to the placenta (eEVTs). Proper differentiation of CTB is essential to ensure adequate blood, oxygen and nutrient delivery throughout pregnancy. Many pregnancy diseases such as preeclampsia (PE) and abnormally invasive placenta (AIP), notably the high grade disorders like *Placenta accreta*, *increta*, *and percreta* are caused by an impaired invasion of EVT into the maternal compartment. However, there is no causative therapy for the above-mentioned pregnancy diseases, resulting in a heavy global burden (2–4).

PE is a pregnancy-related hypertensive disorder characterized by an insufficient trophoblast invasion into the maternal spiral arteries, deregulated proliferation and enhanced apoptosis (5, 6). PE is also a major contributor to perinatal morbidity and mortality (7). The estimated pregnancy rate of PE is 4.5% and this ratio appears to be increasing (3, 4). Despite decades of studies, important information on detailed molecular mechanisms leading to PE is still lacking. There are two types of preeclampsia: the early-onset form (delivery before 34 weeks’ gestation), which is often associated with a high rate of fetal growth restriction (FGR), while the late-onset form (delivery after 34 weeks’ gestation) which is usually not associated with fetal growth restricted complication (8–10). Previous studies reported that the two pathologies are physiologically different disorders, early-onset PE caused by placental factors, but late-onset preeclampsia is considered a maternal disease rather than a placental disease (8). However, in a recent publication, Staff et al. (9) reported in a new hypothesis that all stages of preeclampsia require impaired placental development (9). AIP implies an abnormal placental position/location characterized by direct apposition of placental villi at the border to the myometrium or even by penetrating the myometrial tissue (11–13). This disease is mainly caused by previous cesarean section or operative intervention within the uterine cavity which is associated with a defect in decidualization (13). As the cesarean section rate increases, the incidence of AIP has increased over the past 50 years (14). It is difficult for patients with AIP to strip the entire placenta after delivery, which leads to serious obstetric complications, such as hemorrhages, hysterectomy, and even maternal mortality (15, 16). The molecular mechanisms of invasive placentation in AIP are poorly understood; it might be a combination of primary absence of the decidua or basal plate, abnormal maternal vascular remodelling, and excessive EVT invasion (17)

In summary, PE and AIP are consequences of two opposing trophoblast invasion disorders. Previous studies proved that excessive trophoblast invasion might contribute to the pathogenesis of AIP (17, 18). For PE, it is known that the decline in trophoblast invasion and migration leads to placental hypoperfusion (19). However, the pathophysiology of PE associated with a shallow invasion and on the opposite, the different AIPs associated with accelerated trophoblast invasion, are not fully understood. However, the complicated pathogenesis of PE and AIP may not be explained by a simple single mechanism. For both trophoblast subpopulations, STB and EVT, a strong regulation during placental development in trophoblast proliferation and cell cycle arrest to achieve a well-adjusted balance in placental growth, invasion and aging are necessary. EVTs represent the source of trophoblast cells responsible for invasion into the decidua to arrode maternal vessels. This trophoblast subpopulation needs to be in a balance of proliferating cells proximal of the cell column and the escape from cell cycle before entering the maternal blood system (20). Key molecules and pathways affecting the process of trophoblast proliferation, invasion and cell cycle arrest for preventive and therapeutic strategies are still under debate. However, it is well known that the fusion from CTB to STB induces cell cycle arrest which is called cellular senescence (21). The process of cellular senescence affects trophoblast differentiation and proliferation. Alteration of senescence-related markers on STB may have a relationship with PE and AIP (22–24). Ray J et al. (25) found that *in vitro* differentiation from CTB to STB coincided with a decrease in cyclin E1, which is a G1/S phase cell cycle regulator that can inhibit cell cycle progression (25). Previous studies also reported that the AKT signaling pathway is involved in the invasion process as well as cell migration which have a relationship with the placenta diseases PE and AIP (19, 26, 27).

Recently, we revealed that CCN3 (*nephroblastoma overexpressed*, NOV), a member of the CCN family (28), plays an important role in regulating trophoblast proliferation, migration and invasion (29–32), inducing cell cycle arrest (33). Decreased expression of CCN3 was found in preeclamptic placentas (34) which may lead to an imbalance between trophoblast proliferation and invasion and might contribute to the shallow trophoblast invasion. This hypothesis is supported by our recent investigations that CCN3 induces senescence of a first trimester trophoblast cell line SGHPL-5 *in vitro*, which is accompanied by a cell cycle arrest at G0/G1, and CCN3 increases the expression of cleaved-Notch1/p21 and p16, thereby decreasing cell proliferation (33). Simultaneously, CCN3 seems to promote migration capability by activating focal adhesion kinase (FAK) and AKT kinase (protein kinase B) (33).

Here, we investigated pathological placental tissues of preeclampsia and AIP versus age-matched control tissues to confirm our results derived from trophoblast cell lines and to verify their clinical situation value. The present study aims to test if CCN3 plays a role in *in situ* placental pathologies with shallow or enhanced trophoblast invasion and if the findings correspond to our hypothesis from the *in vitro* investigations (30, 31, 33).

We hypothesize that CCN3 expression is dysregulated in pathological placentas by interfering with the cell cycle machinery and also changing trophoblast cell migration and invasion process through AKT signaling pathway. Thus, maybe *via* CCN3 regulation within the placenta, the etiology of theses placental diseases could be explained, leading to novel therapeutic strategies to interfere with them.

## Materials and Methods

### Human Placental Samples

Placental tissues were obtained from the Department of Gynecology and Obstetrics, University Hospital Essen, Germany, between 2014 and 2019. The respective ethics committee approved the study by obtaining consent forms (No.: 12-5212-BO). Placental tissue was obtained at the time of vaginal delivery or caesarian section. 50 pregnant women were recruited for this study and classified in early (delivery < 34 weeks) and late (delivery > 34 weeks) cases. According to the pathogenesis of early and late PE, we respectively categorized the AIP cases in early and late cases for comparison reasons. We are aware that late AIP represents only a progressive stage and not different pathogenesis of AIP. In contrast to PE, the preterm delivery of the AIP cases is clinically indicated due to maternal discomfort and bleeding.

The following pathological study groups were analyzed: For PE we investigated 25 patients and for AIP 8 patients: early-onset preeclampsia (early PE) [delivery before weeks 34, n = 16 (including N = 10 FGR)], late-onset preeclampsia (late PE) [delivery after weeks 34, n = 9 (including N = 2 FGR)]; early AIP (delivery before weeks 34, N = 1 *accreta*; N = 3 *percreta*); late AIP (delivery after weeks 34, N = 1 *accreta*, N = 1 *increta*, N = 2 *percreta*).

Preeclampsia was diagnosed according to the American College of Obstetricians and Gynecologists (ACOG) (2019) of Hypertensive Disorders in Pregnancy (35). At the time of tissue collection, preeclampsia was defined as an occurrence of hypertension after 20 weeks of gestation with a blood pressure of at least 140/90 mm Hg or blood pressure of at least 160/110 mm Hg or detectable proteinuria measured by dipstick ≥ 1+ (30 mg/dL) according to the guidelines of the International Society for the Study of Hypertension in Pregnancy (36) as well as accompanied by a sFLT-1 (soluble fms-like tyrosine kinase-1)/PLGF (placental growth factor) blood serum level of > 85 in early-PE or > 110 in late PE (37). sFLT-1/PLGF ratio serves as a clinical biomarker for PE (37).

Fetal growth restriction (FGR) was defined as the pregnancies ending with a newborn with a birth weight below the 10th centile and pathological Doppler characteristics (A. umbilicalis or Aa. uterinae) according to the 2019 ACOG Clinical Guidelines for FGR.

AIP was diagnosed during pregnancy by ultrasound measurements according to the criteria defined by Cali et al. (38), in the department of Gynecology and Obstetrics, Prenatal Diagnosis in the University Hospital of Essen Germany, and characterized with the FIGO classification based on intraoperative situation and histological findings. In *Placenta accreta*, the decidua basalis is completely lost or partly so that the trophoblast is directly apposed to the myometrial tissue without invading it. *Placenta increta* is defined by a deep invasion of trophoblast cells into the myometrium and in *Placenta percreta* cases trophoblast cells involve and penetrate the uterine serosa (13). The FIGO recently suggested the following classification: grade 1: Placenta *adherenta* or *accreta*, grade 2: *Placenta increta*, grade 3a: *Placenta percreta* limited to the uterine serosa, grade 3b: *Placenta percreta* with urinary bladder invasion and grade 3c: *Placenta percreta* with the involvement of pelvic tissue/organs (13). Most cases of AIP are *Placenta accreta* (70%), followed by *Placenta increta* in 15% and *Placenta percreta* in 11% (11). Histological analysis was performed in the Institute for Pathology, University Hospital Essen, Germany. Patient’s characteristics of all study groups are summarized in **Table 1**. For AIP cases we investigated the following grades (grade 1: *accreta*, n = 2; grade 2: *increta*, N = 1) and grade 3: *percreta*: 3a: N = 2, 3b: N = 1, 3c: N = 2). **Supplementary Table 1** shows the pregnancy course and FIGO classification of the AIP cases. Since we observe a similar and uniform pattern of gene expression of the analyzed genes in the study independent on the grades in AIP cases (*accreta*, *increta*, or *percreta*), we combined the data of all AIP cases, separated in early and late AIP, for qPCR and western blot analysis independent from pathological stages classified by FIGO.

**Table 1:** Characteristics of the study population.

	Early controlsN = 7	Early PEN = 16	Late controlsN = 10	Late PEN = 9	Early AIPN = 4	Late AIPN = 4
**Nulliparous, no. (%)**	2 (29%)	9 (56%)	2 (20%)	8 (89%)	1 (25%)	0 (0%)
**Maternal age at delivery (years), median (IQR)**	32 (28.0–43.0)	32.5 (26.25–35.75)	28.5 (27.75–32.75)	33 (29.5–36.5)	37 (33.0–38.75)	36.5 (34.5–40.0)
**Gestational age at delivery (days), median (IQR)**	224 (211.0–232.0)	203.5 (191.0–226.0)	263.5 (256.5–275.0)	253 (244.5–256.0)	211 (200.3–222.5)	259 (252.3–274.8)
**Cigarette smoking, no. (%)**	2 (29%)	1 (6%)	0 (0%)	0 (0%)	0 (0%)	0 (0%)
**Caucasian ethnicity, no. (%)**	7 (100%)	14 (88%)	9 (90%)	9 (100%)	3 (75%)	4 (100%)
**Systolic Blood pressure, mmHg, median (IQR)**	116 (100.0–125.0)	151**** (142.0–168.0)	129.5 (114.8–137.0)	150* (135.0–173.0)	109 (105.3–125.5)	117 (104.5–129.5)
**Diastolic blood pressure, mmHg, median (IQR)**	66 (56.00-75.0)	94.0**** (89.0–100.0)	72 (58.75–82.25)	99** (89.0–111.0)	56.5 (54.5–66.75)	58.5 (51.75–73.5)
**Proteinuria, mg/24 hour, median (IQR)**	nm	1070 (498–4795)	nm	410 (315-1248)	nm	nm
**sFLT-1/PlGF ratio, median (IQR)**	6.176 (3.036-11.50)	951.2** (472.2–1631)	3.63 (1.6–79.0)	116.3* (85.08–372.9)	nm	nm
**Birth weight, g, median (IQR)**	2050 (1354-2330)	1040* (665.0–1374)	3155 (2678–3413)	2760 (2,418–3,305)	1765 (1393–1950)	2780 (2575–3431)
**Birth weight, percentile, median (IQR)**	65 (28.75–71.25)	8** (1.65–23.75)	50 (22.4–84.25)	50 (19.0–84.0)	81 (27.7–92.75)	35 (30.25–47.25)
**Cesarean section, no. (%)**	7 (100%)	15 (94%)	8 (80%)	9 (100%)	3 (75%)	3 (75%)
**Pregnancy BMI before birth** **median(IQR)**	29 (26.5–42.0)	27.0 (24.0–37.0)	30 (27.0–34.0)	32.5 (30.0–38.75)	30 (18.0–48.0)	34.5 (32.25–42.75)
**Fetal sex (%)**						
**Male**	50%	35%	50%	67%	67%	25%
**Female**	50%	65%	50%	33%	33%	75%

*p < 0.05; **p < 0.01; ****p < 0.0001; nm, not measured.

The control group for PE and AIP includes early controls (26-34 weeks, n = 7) and late controls (34-40 weeks, n = 10). Controls were defined as pregnancies without any characteristics of placental disorders from the AIP spectrum. Late controls only included uneventful pregnancies without diabetes, fetal abnormalities or infections. We are aware that preterm deliveries are commonly not uneventful pregnancies and therefore, we strictly used placentas of pregnancies without any characteristics of the AIP and PE but with other complications (n = 2 placental abruption, n = 2 maternal indications (malignancy; threatening uterine rupture), n = 1 amnioninfection syndrome, n = 1 severe FGR without PE and n = 1 *Placenta praevia* with bleeding) as early controls (see patient characteristics in **Supplementary Table 2**). All early control patients of PE and AIP had a cesarean section. Nevertheless, using gestational-matched controls is absolutely necessary because changes in gene expression pattern during trophoblast differentiation correlate to the gestational age (39).

### Placental Dissection

For immunofluorescence, placental chorionic villous tissue was cut from the maternal side of the placenta between the umbilical cord and the outer border of the placenta, including the decidua, and washed twice briefly by sterile phosphate buffer solution (PBS). In AIP cases, the placental tissue with the deepest placental penetration was chosen for analysis and was collected including surrounding tissue (decidua, myometrium, uterine serosa, broad ligament tissue) by the identical person in all cases who also performed the operation. All tissue samples were fixed in 4% formalin overnight before standard processing to obtain paraffin-embedded sections.

For RNA and protein isolation, only placental chorionic tissue without decidua was collected. Tissues were frozen in liquid nitrogen and stored at −80°C until the extraction of RNA and protein samples.

### RNA Extraction, cDNA Synthesis, and Quantitative PCR

Total RNA was extracted from 20∼30 mg frozen samples of human placenta with the E.Z.N.A Total RNA Kit (Omega Bio-tek, Norcross, GA, USA) according to the manufacturer’s protocol. Total placental RNA samples (1 µg) were DNase-digested and reverse transcribed as described previously (34). Gene expression of CCN3, p16, p21, and Cyclin D1 was quantitated using the qPCR Master Mix SYBR Green (Affymetrix, Santa Clara, USA) and analyzed using an ABI Prism 7300 sequence detector (Applied Biosystems, Foster City, USA). After intensive testing of different genes as housekeepers to find an appropriate endogenous reference measuring mRNA amount, we decided to choose HPRT1 (hypoxanthine phosphoribosyltransferase 1) as an internal reference control which showed the most stable expression already shown in other publications (40, 41). The used primers and sequences are listed in **Table 2**. For a detailed description of the PCR parameters used, refer to our previous publication (34). Ten-fold dilutions of purified PCR products were used as standards (CCN3/p16/Cyclin D1 start from at 100 fg to 0.01 fg, p21 start from 1 pg to 0.1 fg, HPRT1 start from 10 fg to 0.001 fg). The quantity of cDNA in each sample was normalized to the HPRT1 cDNA. We tested the following experimental groups: early control, n = 7, early PE, n = 16, early AIP, n = 4, late control, n = 10, late PE, n = 9, late AIP, n = 4.

**Table 2:** Sequences of primers.

Gene	NCBI accession number	Primer Sequence (5’ → 3’)	Product length
CCN3	NC_000008.11	for: CACGGCGGTAGAGGGAGATArev: GGGTAAGGCCTCCCAGTGAA	251 bp
p16	XM_011517676	for: CATGGAGCCTTCGGCTGACrev: GGCCTCCGACCGTAACTATT	120 bp
Cyclin D1	NM_053056.2	for: GCATGTTCGTGGCCTCTAAGrev: CGTGTTTGCGGATGATCTGT	228 bp
p21	NM_001291549.2	for: GCGATGGACTTCGACTTTGrev: CAGGTCCACATGGTCTTCCT	198 bp
HPRT1	NM_000194.2	for: GACCAGTCAACAGGGGACATrev: CCTGACCAAGGAAAGCAAAG	132 bp

### Western Blot

Protein extracts were prepared as described previously (34). Protein samples (20 μg) were separated on 4%–20% polyacrylamide gel (Amersham Biosciences, Piscataway, NJ, USA). Next, proteins were transferred onto PVDF membranes and incubated at 4°C overnight with the primary antibodies. The following primary antibodies were used according to **Table 3**. The secondary antibody was incubated for 1 hour at room temperature (goat anti-rabbit HRP, Pierce, #1858415). Detection was achieved with the ECL chemiluminescence kit (Amersham Biosciences) according to the protocol and analyzed using the Chemidoc XRS+ imaging system (BioRad, Feldkirchen, Germany). Densitometric analysis of single protein bands were performed by Image J2 x (Rawak Software Inc., Germany), and then the protein expression levels were normalized to actin expression. For normalization purposes, each signal’s value was normalized to a same “internal control” sample which was run on each blot.

**Table 3:** Primary and secondary antibodies used for western blotting.

Primary antibody				Secondary antibody
Primary antibody	Host	Company	concentration	concentration
CCN3	rabbit monoclonal	Abcam (ab137677)	1:500	1:100.000
p16	rabbit monoclonal	Abcam (ab108349)	1:250	1:1000
p21	rabbit monoclonal	Cell Signaling (#2947S)	1:750	1:2500
Cyclin D1	rabbit monoclonal	Abcam (ab13417)	1:150.000	1:1000
β-Actin	Mouse Monoclonal-β Actin Peroxidase	Sigma (#015M4866V)	1:200.000	–
p53	rabbit monoclonal	Cell Signaling (#9284S)	1:1000	1:5000
cleaved Notch-1	rabbit monoclonal	Cell Signaling (#4147T)	1:250	1:3500
Cyclin E1	rabbit monoclonal	NOVUS (NBP2-67443)	1:750	1:5000
pRb	rabbit monoclonal	Cell Signaling (#9307S)	1:1000	1:4000
pAkt	rabbit monoclonal	Cell Signaling (#4060S)	1:1000	1:5000
Akt	rabbit monoclonal	Cell Signaling (#9272S)	1:1000	1:5000
pFAK	rabbit monoclonal	Cell Signaling (#4060S)	1:1000	1:2500
FAK	rabbit monoclonal	Santa Cruz (sc-775)	1:1000	1:2500
pmTOR	rabbit monoclonal	Cell Signaling (#2974S)	1:250	1:2500
mTOR	rabbit monoclonal	Cell Signaling (#2972)	1:750	1:2500

### Immunofluorescence Staining

Unstained tissue sections were deparaffinized and antigen retrieval was performed in a citrate buffer at 100°C for 40 min followed by permeabilization with 0.3% Triton X-100 in PBS for 20 min. Non-specific sites were blocked by incubation in 0.5% BSA in PBS for 20 min and autofluorescence was blocked by Sudan Black (Dianova, Hamburg, Germany). Primary and secondary antibodies used are listed in **Table 4**. Incubation with the primary antibody was performed overnight at 4°C, and incubation with the respective secondary antibody for 1h at room temperature. All samples were counterstained with 4’, 6-diamidin-2-phenylindol dihydrochloride (DAPI, 1µg/ml, Sigma Aldrich, St. Louis, USA) for 15 min at room temperature. Negative controls were performed by omitting the primary antibody. Slides were covered with Mowiol (Roth, Karlsruhe, Germany) and examined using a confocal fluorescence microscope (Leica SP5) and the software analysis program LAS AF (Leica). A minimum of three placental tissue samples for each condition was investigated.

**Table 4:** Primary and secondary antibodies used for immunohistochemistry.

Primary antibody	Host (clone)	Company
HLA-G	mouse monoclonal (4H84)	Antibodies online (ABIN192401)
CK-7 (cytokeratin 7)	mouse monoclonal	Novus biologicals (NBP1-22539)
CCN3	rabbit monoclonal	Abcam (ab137677)
p16	rabbit monoclonal	Abcam (ab108349)
Cyclin D1	rabbit monoclonal	Abcam (ab74646)
p21	rabbit monoclonal	Cell Signaling (#2947)
**Secondary antibody**	**Host (clone)**	**Company**
Alexa Fluor 488	donkey anti rabbit IgG	Life Technologies (R37118)
Cy3	goat anti mouse	Life Technologies (A10521)

### Statistical Analysis

Data of western blot and qPCR were not Gaussian distributed. Thus, the Mann-Whitney test was used for non-parametric independent two-group comparisons to compare the results of gestational- matched control groups and preeclamptic as well as AIP cases. Statistical analyses were performed using GraphPad Prism 6.0 (GraphPad Software Inc., La Jolla, San Jose, CA, USA). Data are either presented in mean ± standard error. For all statistical tests, a probability value (p-value) of 0.05 or less was indicated with *p < 0.05, **p < 0.01, ***p < 0.001, and ****p < 0.0001. Outliers were detected performing Grubbs’ test (https://www.graphpad.com/quickcalcs/Grubbs1.cfm).

## Results

### Clinical Features of Pregnant Women

**Table 1** shows the clinical features of pregnant patients. Placental tissues from 50 pregnant women (N = 7 early control; N = 10 late control; N = 16, early-onset PE; N = 9, late-onset PE; N = 4 early AIP; N = 4 late AIP) showed no significant difference in the maternal age, ethnicity, cesarean section rate and maternal BMI (p > 0.05) among the groups. The systolic blood pressure in early PE patients (p < 0.0001), and in late PE (p < 0.05) and the diastolic blood pressure in early PE (p < 0.0001) and late PE (p < 0.01) were significantly different compared to controls. The level of the sFLT-1/PlGF ratio is significantly increased in early and late PE compared to gestational age-matched controls. The birth weight and birth weight percentile are significantly lower in early PE (p < 0.05) while there is no difference in the late PE group. All these parameters did not show a significant difference in patients with AIP.

### Transcript Expression of CCN3, p16, p21, and Cyclin D1 in Preeclamptic Placenta and AIP

**Figure 1:**
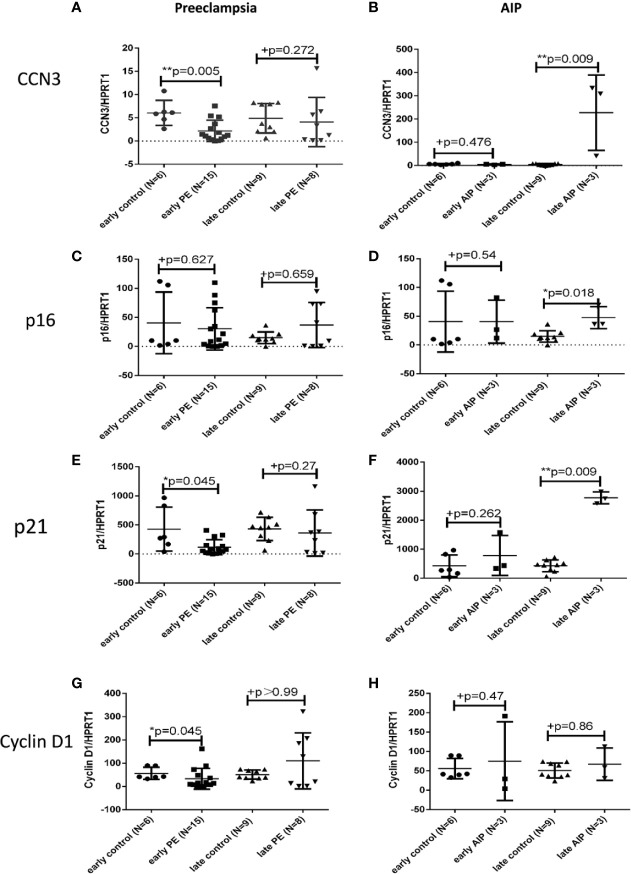
Transcript expression of CCN3, p16, p21, and Cyclin D1 in preeclamptic and AIP placentas. Analysis of the mRNA expression of CCN3 **(A, B)**, p16 **(C, D)**, p21 **(D, E)**, and Cyclin D1 **(G, H)** in the placentas of early control (N = 7), late control (N = 10), early PE (N = 16), late PE (N = 9), early AIP (N = 4), and late AIP (N = 4) placentas. The relative mRNA levels were tested by qRT-PCR. The transcript levels of examined genes were compared after normalization to HPRT1. Data represent means ± SD. *p < 0.05 and **p < 0.01, significantly up-/down-regulated compared to controls. +p > 0.05 indicates that there is no significant differences between the two and matched groups. The box blots showed that the mRNA of CCN3 **(A)**, p21 **(E)**, and Cyclin D1 **(G)** were significantly decreased in early PE compared to early control while there is no significant difference in late PE compared to late control. The mRNA expression of p16 **(C)** was not different in both groups. **(B, D, F)** show that the CCN3, p16 and p21 mRNA levels were significantly increased in late AIP while no difference in early AIP compared to gestational age-matched controls was shown. The Cyclin D1 mRNA level was not significantly different in both AIP groups **(H)**.

Quantitative RT-PCR was used to detect the mRNA levels of CCN3, p16, p21, and Cyclin D1 in PE, and AIP placentas compared to gestational age-matched controls. In early-onset PE, CCN3 mRNA expression was significantly lower expressed (p = 0.005) (**Figure 1A**). In contrast, there was no significant difference between late PE and late control group (**Figure 1A**). The marker gene for cell cycle control p16 is not significantly different from gestational age-matched controls in early and late PE (**Figure 1C**). The transcript levels of p21 were significantly lower expressed in early but not late preeclamptic placentas (p = 0.045) (**Figure 1E**). The expression of Cyclin D1 was lower expressed in the early PE group (p = 0.045) (**Figure 1G**).

In contrast to early PE, the expression of CCN3 in early AIP placentas was not significantly different between pathological and control placentas (**Figure 1B**). However, in late AIP cases, the transcript levels of CCN3 (p = 0.009), p16 (p = 0.018) and p21 (p = 0.009) were significantly higher expressed compared to the late control group (**Figures 1B, D, F**). However, p21 was not differently expressed in early AIP group. The Cyclin D1 mRNA was not different in both early and late AIP groups (**Figure 1H**).

### Protein Expression of CCN3, p16, p21, and Cyclin D1 in Preeclamptic Placenta and AIP

**Figure 2:**
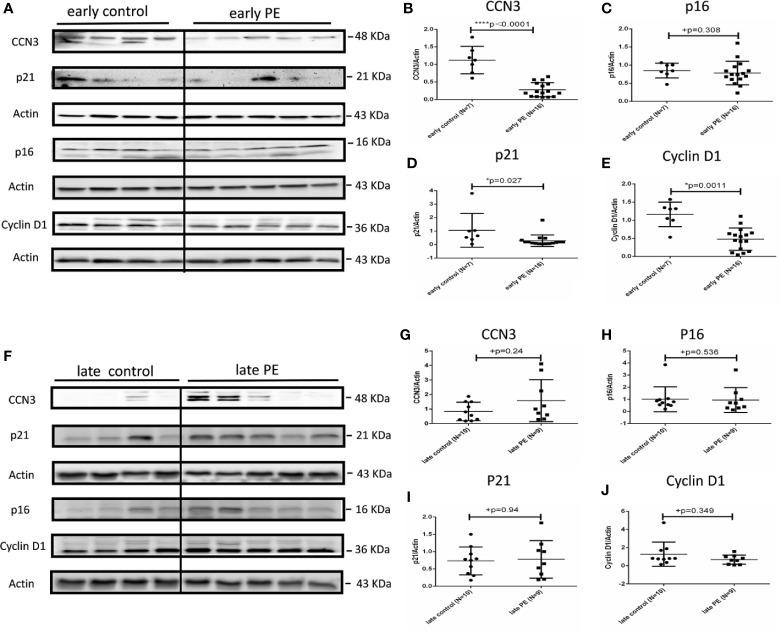
Protein expression of CCN3, p16, p21, and Cyclin D1 in early and late preeclamptic placentas. **(A)** Representative western blot of CCN3, p16, p21, and Cyclin D1 protein expression in the early control group (N = 7) and early PE group (N = 16). **(B–E)** CCN3, p16, p21, and Cyclin D1 protein levels are normalized to Actin expression and further on normalized to the same sample as internal control which runs on each gel. The expression of CCN3, p21 and Cyclin D1 was significantly lower than controls, while p16 was not significantly different in the early PE group. **(F)** Representative western blot of CCN3, p16, p21, and Cyclin D1 protein expression in the late control (N = 10) and late PE (N = 9). **(G–J)** The expression of CCN3, p21 and Cyclin D1 was significantly lower compared to the control group while the p16 was not significantly different in the early PE group. Protein expression of CCN3, p21, p16, and Cyclin D1 was not significantly different between late PE and late control groups. Data represent means ± SD. *p < 0.05 and ****p < 0.0001, significantly up-/down-regulated compared to the control. +p ≥ 0.05 indicates that there is no significant difference between the groups.

To confirm the results on protein level, we next analyzed the protein expression of cell cycle regulator proteins by Western blot analysis in PE, AIP and controls. CCN3 protein expression was significantly lower expressed (p < 0.0001) in early PE group compared to gestational age-matched controls (**Figures 2A, B**).

The expression of p16 was not significantly different in early PE (**Figures 2A, C**). However, p21 and Cyclin D1 protein levels were significantly lower expressed in early PE group (p = 0.027 and p = 0.0011) (**Figures 2A, D, E**). CCN3, p16, p21, and Cyclin D1 showed no significant difference between late PE and controls (**Figures 2F–J**).

**Figure 3:**
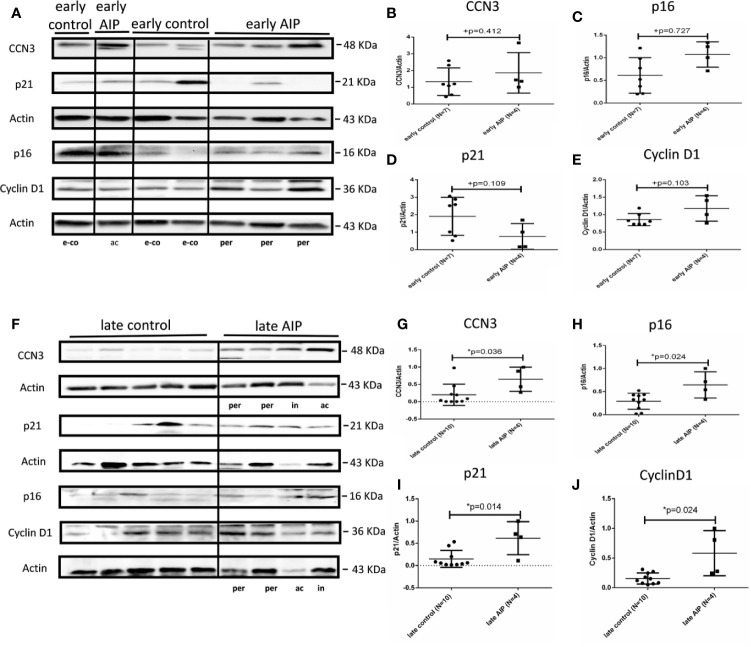
Protein expression of CCN3, p16, p21, and Cyclin D1 in AIP placentas. **(A)** Representative western blot of CCN3, p16, p21, and Cyclin D1 protein expression in the early control group (N = 7) and early AIP group (N = 4), e-co represents early control, ac and per represent *Placenta accreta* and *Placenta percreta* samples. **(B–E)** CCN3, p16, p21, and Cyclin D1 protein levels were normalized to Actin and further on normalized to a same sample run on each gel. CCN3, p16, p21, and Cyclin D1 protein levels are not significantly different between early control and early AIP. **(F)** Representative western blot of CCN3, p16, p21, and Cyclin D1 protein expression in the late control (N = 10) and late AIP (N = 4). ac, in and per represent *Placenta accreta*, increta, and *Placenta percreta* samples. **(G–J)** The expression of CCN3, p21 and Cyclin D1 was significantly increased in the late AIP. Data represent means ± SD. *p < 0.05, significantly up-/down-regulated compared to the control. +p ≥ 0.05 indicates that there is no significant difference between the groups.

In early AIP, CCN3, p16, p21, and Cyclin D1 proteins expression were not significantly different from those in the control group (**Figures 3A–E**). However, in late AIP group, CCN3, p16, p21 and CyclinD1 proteins expression were significantly increased compared to gestational age-matched controls (**Figures 3B, F–J**).

In summary, our study showed that CCN3 expression on mRNA and protein level is conversely regulated in PE and AIP cases, with a down-regulation in early-onset PE and an up-regulation in late AIP placentas (see also **Supplementary Table S3** for comparison of mRNA and protein data). This regulation pattern is also found for p21 and partly for Cyclin D1 whereas p16 is only significantly increased in late AIP, but not changed in early PE. In contrast, in late PE and early AIP CCN3, p16, p21, and Cyclin D1 were not differently expressed.

### Protein Expression of Cleaved Notch-1 and p53 in Preeclamptic Placenta and AIP

**Figure 4:**
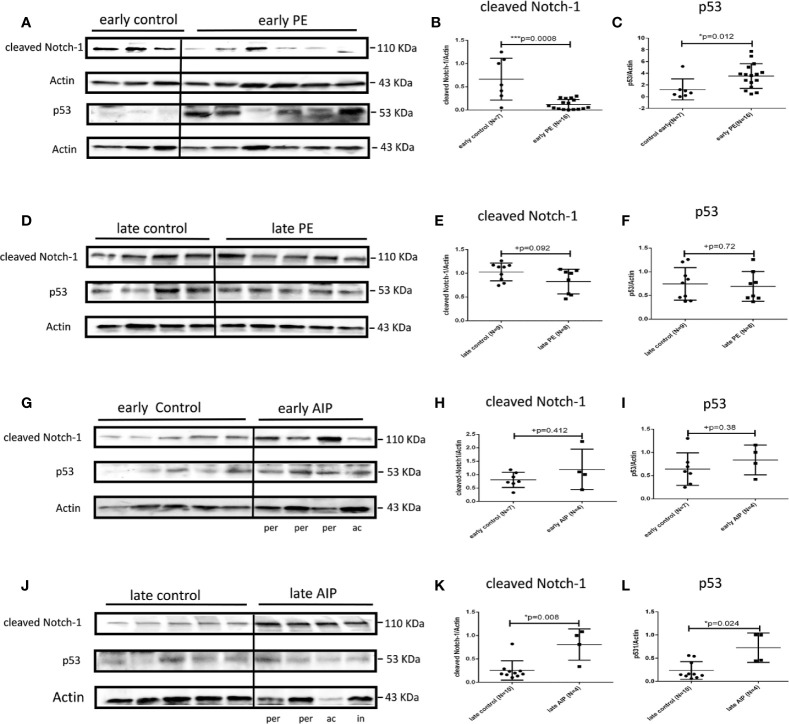
Protein expression of cleaved Notch-1 and p53 in preeclamptic placentas and AIP placentas. **(A)** Representative western blot results of cleaved Notch-1 and p53 protein expression in the early control (N = 7) and early PE (N = 16). **(B, C)** The expression of cleaved Notch-1 was significantly lower while p53 was significantly increased in the early PE group. **(D)** Representative western blot of cleaved Notch-1 and p53 protein expression in the late control (N = 10) and late PE (N = 9). **(E, F)** Protein expression of cleaved Notch-1 was not significantly different between late PE and late control group**. (G)** Representative western blot results of cleaved Notch-1 and p53 protein expression in the early control group (N = 7) and early AIP group (N = 4), e-co represented early control, ac and per represent *Placenta accreta* and *Placenta percreta* samples. **(H, I)** Cleaved Notch-1 and p53 protein levels are not significantly different between the early control group and early AIP. **(J)** Representative western blot of cleaved Notch-1 and p53 protein expression in the late control (N = 10) and late AIP (N = 4), l-co represented late control, ac, in and per represent *Placenta accreta*, *increta* and *Placenta percreta* samples. **(K, L)** The expression of cleaved Notch-1 and p53 was significantly increased in the late AIP group. The cleaved Notch-1 and p53 protein levels were normalized to Actin and further on normalized to the same sample run on each gel. Data represent means ± SD. *p < 0.05 and ***p < 0.001, significantly up-/down-regulated compared to the control. +p ≥ 0.05 indicates that there is no significant difference between the groups.

In early PE group, cleaved Notch-1, which regulates p21 (42), was significantly lower expressed compared to early controls (p = 0.0008) (**Figures 4A, B**), whereas the expression of p53, a regulator of trophoblast cell apoptosis (43) was significantly increased (p = 0.012) compared to the control group (**Figures 4A, C**). There were no significantly different expression levels of cleaved Notch-1 and p53 in late PE and early AIP (**Figures 4D–I**). However, in late AIP both cleaved Notch-1 and p53 protein expression were significantly increased (**Figures 4J–L**).

### Protein Expression of pRb, Cyclin E1, pFAK, pAkt, and pmTOR in Early PE Placenta and Late AIP

Since we found significant differences in the cell cycle regulator genes (cleaved Notch-1, p21, p16, p53, Cyclin D1) only in early PE and late AIP placentas, we focused on these placentas for further analyses of pRb, Cyclin E1, and the signaling kinases FAK, Akt and mTOR which are known to be involved in the CCN3 signaling pathways in the trophoblast (31, 33).

In early PE, the expression of pRb and Cyclin E1 was significantly increased compared to gestational age-matched controls (**Supplementary Figures S1A–C**), whereas pFAK/FAK, pAkt/AKT and pmTOR/mTOR protein levels were significantly decreased in early PE (**Supplementary Figures S1A, D–F**). In contrast to early PE in late AIP, pRb protein expression was significantly down-regulated (**Supplementary Figures S2A, C**), and pFAK, pAKT and pmTOR protein expression (**Supplementary Figures S2A, D–F**) was significantly up-regulated. Cyclin E1 expression showed a trend in a decrease in the late AIP group, but a statistical difference compared to controls could not be verified (**Supplementary Figures S2A, B**).

### Immunolocalization of CCN3, p16, p21, and Cyclin D1 in EVT Cells in Early Preeclamptic Placenta and Late AIP

In former studies (29–32, 34) we showed that CCN3 was co-expressed in villous STB and in interstitial EVT cells of normal placentas—pointing to an association with senescence, migration, and invasion properties. Therefore, we here analyzed the localization of CCN3, p16, p21, and Cyclin D1 in STB and EVT cells of early preeclamptic and late AIP placentas, those pathological placentas which already revealed differences in the expression on mRNA and protein level (see above), with a focus on EVT cells characterized by HLA-G+ which were deeply invaded into the decidua.

**Figure 5:**
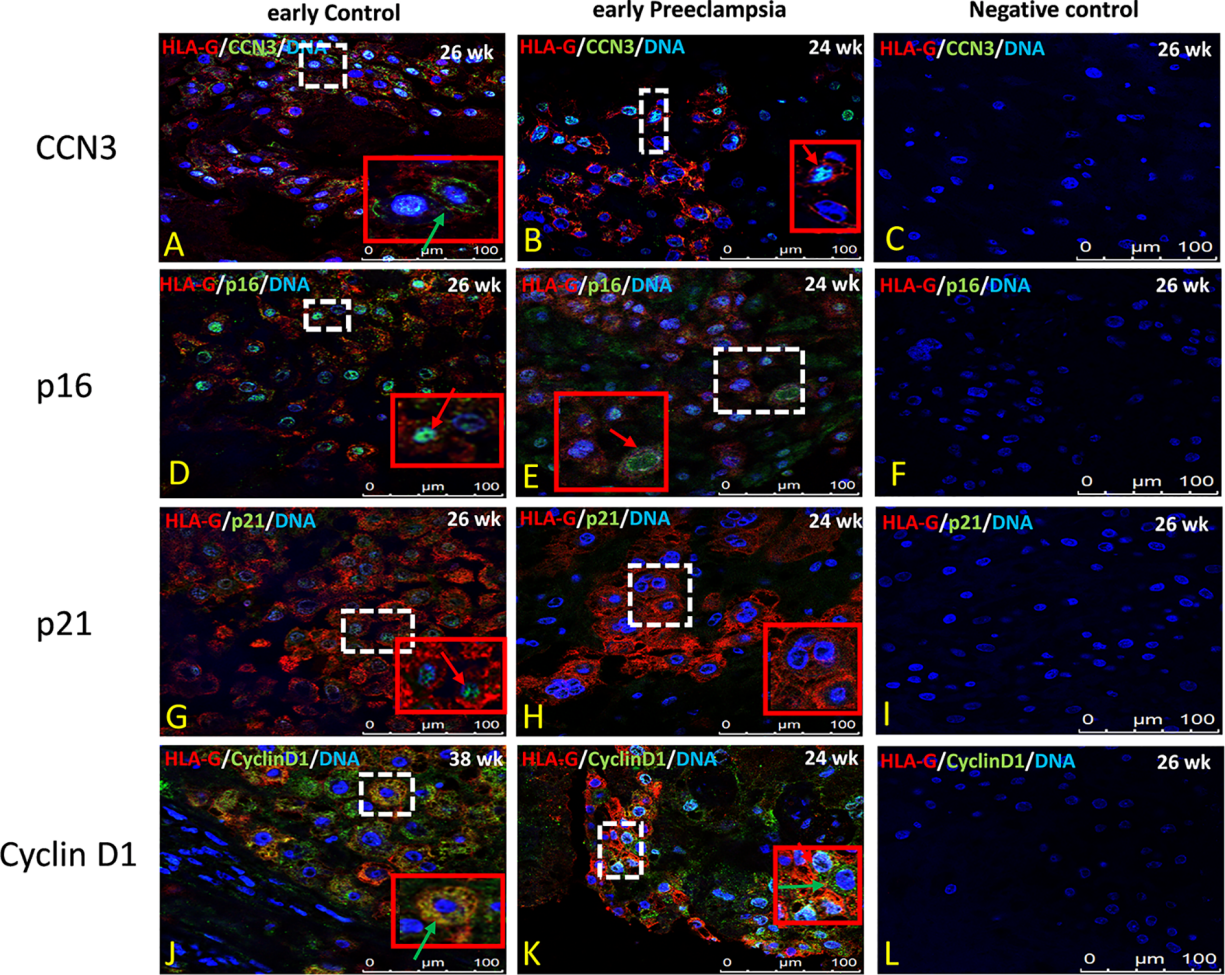
Immunolocalization of CCN3, p16, p21, and Cyclin D1 in EVT cells of early preeclamptic placenta. Double immunolabelling of CCN3 **(A, B)**, p16 **(D, E)**, p21 **(G, H)**, and Cyclin D1 **(J, K)**, in green, with the EVT marker HLA-G, in red; blue, DAPI, respectively. Red arrow, nuclear expression; green arrow, membrane/cytoplasmic expression. Negative controls were performed by omitting the primary antibody **(C, F, I, L)**. Scale bar represents: **(A–L)**, 100 μm. The red box is an enlargement of the white dotted box.

**Figure 6:**
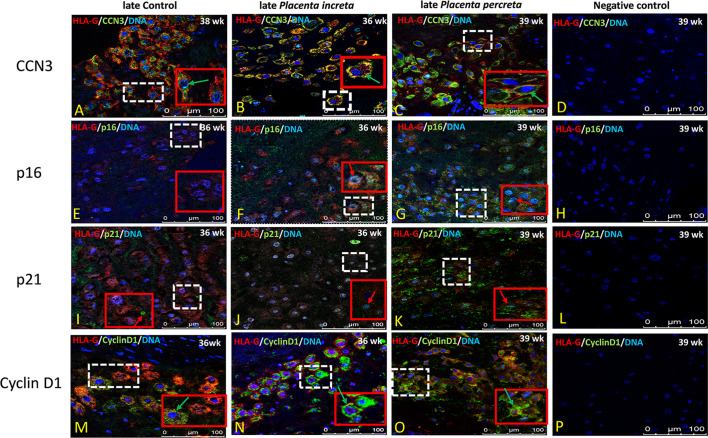
Immunolocalization of CCN3, p16, p21, and Cyclin D1 in EVT cells of late AIP. Double immunolabelling of CCN3 **(A–C)**, p16 **(E–G)**, p21 **(I–K)**, and Cyclin D1 **(M–O)**, in green, with HLA-G, in red; blue, DAPI, respectively. Red arrow, nuclear expression; green arrow, membrane/cytoplasmic. Negative controls were performed by omitting the primary antibody **(D, H, L, P)**. Scale bar represents: **(A–P)**, 100 μm. The red box is an enlarged part of the white dotted box.

Compared to early control placenta tissue (**Figure 5A**), CCN3 immunofluorescence was barely detectable as cytoplasmic staining of EVTs in early PE placentas (**Figure 5B**). However, in PE, CCN3 expression in the nucleus seems not significantly different (red arrow in **Figures 5A, B**). In contrast, compared to late controls (**Figure 6A**), in *Placentas increta* (**Figure 6B**) and *percreta* (**Figure 6C**) tissues, CCN3 showed a strong membrane and cytoplasmic staining (green arrow) for CCN3 in EVTs.

Intense staining of p16 in control placentas was detected in the nucleus of EVT cells (**Figure 5D**). This signal is weaker in EVT nuclei of early preeclamptic placentas (**Figure 5E**). Interestingly compared to the control (**Figure 6E**), in late AIP placentas, shown here for both, *Placenta increta* and *percreta* (**Figures 6F, G**), p16 is strongly expressed both in the cytoplasm and the nucleus in a diffuse pattern of EVT cells.

The localization and staining intensity of p21 in EVT cells is comparable to that of p16. The nuclei in EVT cells of the control placentas showed a strong immunostaining for p21 (**Figure 5G**). Nearly no p21 positive EVT nuclei appeared in placental tissue from PE patients (**Figure 5H**). Compared to late control group (**Figure 6I**), in late AIP we revealed weaker p21 expression in Placenta increta (**Figure 6J**) and a robust p21 expression in EVT cells in *Placenta percreta* (**Figure 6K**).

In the control group, Cyclin D1 is expressed only in the cytoplasm (**Figure 5J**), while in early PE,Cyclin D1 is strongly expressed in the nucleus and weaker in the cytoplasm (**Figure 5K**).

In contrast, in late AIP, intense Cyclin D1 immunolabeling is found in the cytoplasm of EVTs clearly accumulated around the nucleus but only weakly seen in the nucleus (**Figures 6M–O**).

Negative controls were performed by by omitting the primary antibody see **Figures 5C, F, I, L** and **6D, H, L, P**.

### Immunolocalization of CCN3, p16, p21, and Cyclin D1 in Villous Trophoblast Cells of Preeclamptic Placenta and AIP

**Figure 7:**
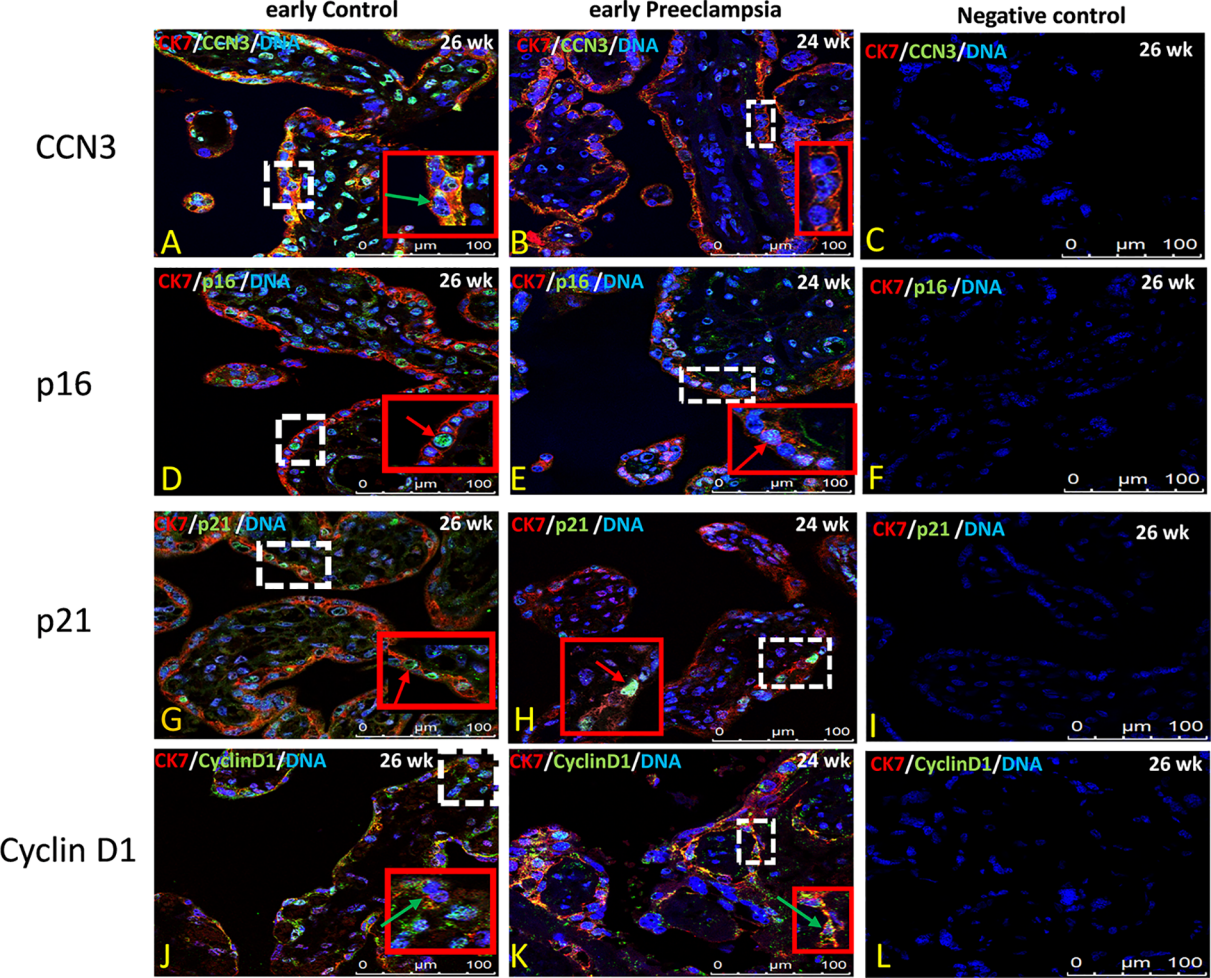
Immunolocalization of CCN3, p16, p21, and Cyclin D1 in villous trophoblast cells of preeclamptic placenta. Double immunolabelling of CCN3 **(A, B)**, p16 **(D, E)**, p21 **(G, H)**, and Cyclin D1 **(J, K)**, in green, with the trophoblast marker CK-7, in red; blue, DAPI, respectively. Red arrow, nucleus expression; green arrow, membrane/cytoplasmic expression. Analysis of representative early control and early preeclamptic placental sections. Negative controls were performed by omitting the primary antibody **(C, H, L, P)**. Scale bar represents: **(A–L)**, 100 μm. The red box is an enlarged part of the white dotted box.

**Figure 8:**
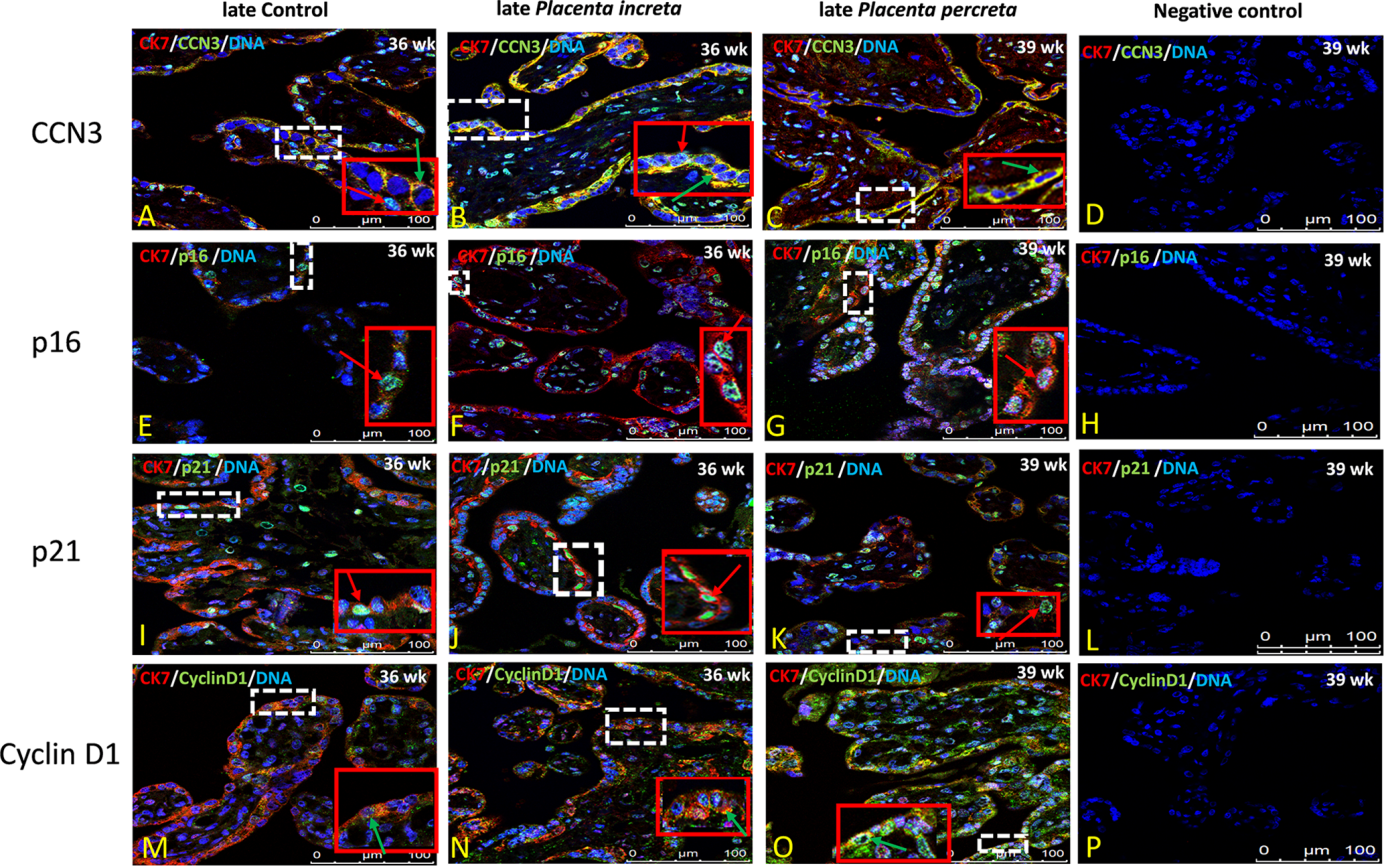
Immunolocalization of CCN3, p16, p21, and Cyclin D1 in villous trophoblast cells of AIP. Double immunolabelling of CCN3 **(A–C)**, p16 **(E–G)**, p21 **(I–K)**, and Cyclin D1 **(M–O)**, in green, with the trophoblast marker CK-7, in red; blue, DAPI, respectively. Red arrow, nuclear expression; green arrow, membrane/cytoplasmic expression. Negative controls were performed by omitting the primary antibody (D/H/L/P). Scale bar represents: **(A–P)**, 100 μm. The red box is an enlarged part of the white dotted box.

The villous trophoblast consists nearly completely of STB, whereas the underlying CTB is already fused into STB at the pregnancy stages investigated. Compared to early control group (**Figure 7A**), in early PE placentas (**Figure 7B**), expression of CCN3 protein was almost absent in the STB analyzed by double immunolabelling with the STB marker cytokeratin 7 (CK-7). Compared to late control group (**Figure 8A**), CCN3 showed a strong membrane and cytoplasmic staining (green arrow) and weak nuclear staining (red arrow) in the STB in the late AIP tissues (**Figures 8B, C**), which is similar to the expression in EVTs (**Figures 5A, B** and **6A–C**).

P16 immunolabelling was mainly found in the nucleus. Compared to late controls (**Figure 8E**), tissue sections from AIP (**Figure 8F, G**) display a very strong nucleus expression signal compared. Compared to controls (**Figure 7D**) p16 staining was obviously decreased in early PE group (**Figure 7E**).

Double immunolabelling of p21 with CK-7 showed that p21 is expressed in the nucleus in STB cells. Compared to controls (**Figure 7G**), sparsely distributed single p21-positive STB appeared in placental tissue from early PE patients (**Figure 7H**), which was lower expressed, whereas compared to late control group (**Figure 8I**), p21 was increased in STB cells of the AIP phenotype (**Figures 8J, K**).

Compared to late control group (**Figure 8M**), Cyclin D1 is strongly expressed in the cytoplasm and in the nucleus of STB in late AIP (**Figures 8N, O**), especially in *Placenta percreta* (**Figure 8O**). Compare to early control group (**Figure 7J**), in early PE placentas Cyclin D1 is expressed also in the cytoplasm and the nucleus; it seems lower expressed in the PE group (**Figure 7K**).

Negative controls were performed by by omitting the primary antibody see **Figures 7C, F, I, L** and **8D, H, L, P**.

### Summary and Conclusions

**Figure 9:**
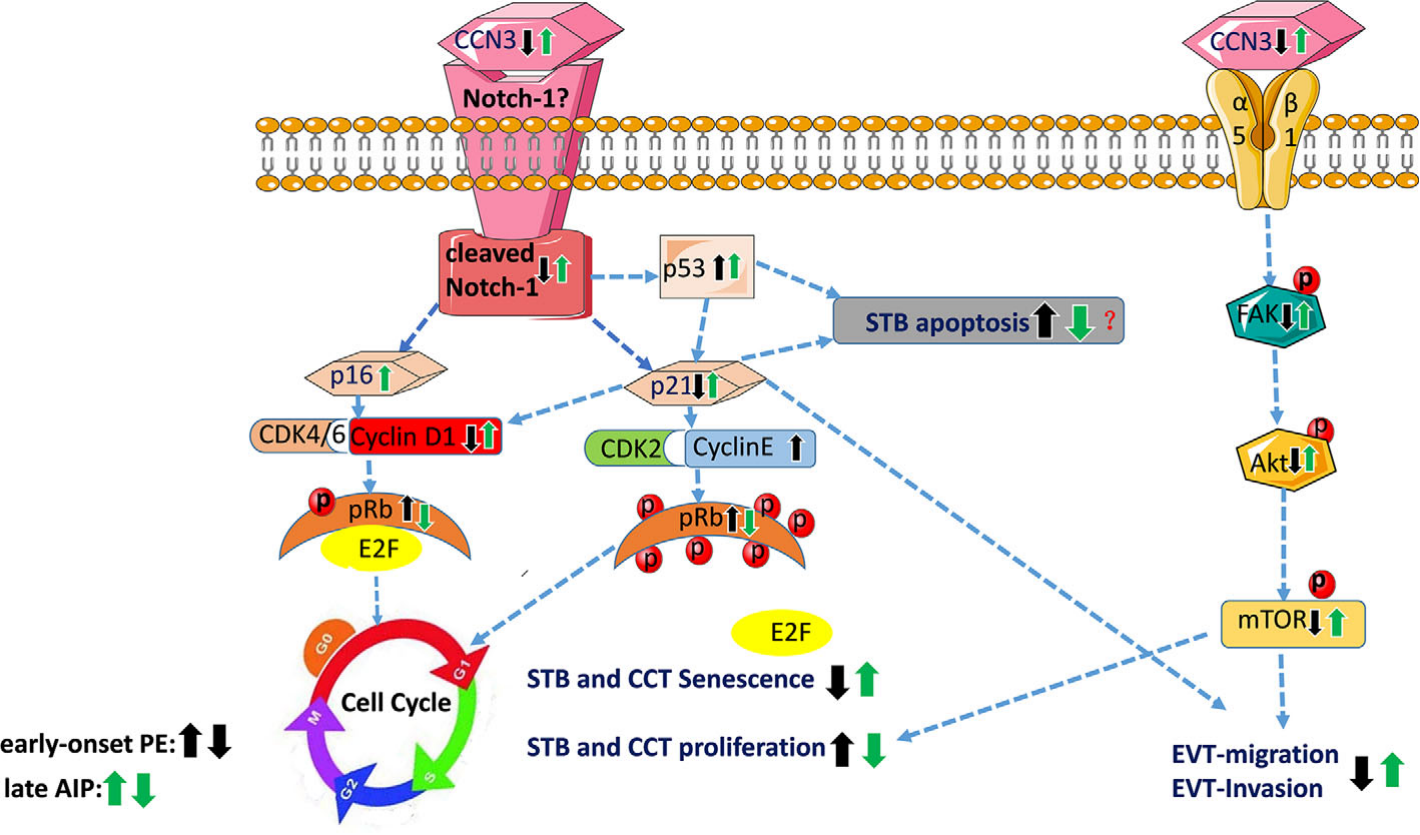
Schematic overview of proposed CCN3-mediated signaling pathways in placental diseases PE and AIP. This figure summarizes the results of this study and showed the proposed signaling pathway of CCN3 in placentas of PE and AIP diseases. In early-onset preeclamptic placentas, CCN3 may promote STB and CCT cell cycle progression *via* p21/Cyclin E1/pRB and FAK/Akt/mTOR pathway. We speculate that the invasion and migration capacities of the EVT may be decreased by subsequently inhibiting FAK/Akt pathway and cleaved Notch/p21 signaling. Increased p53 expression can induce cell apoptosis and decreased p21 that also inducing cell apoptosis, which may result in PE. However, in the late AIP group, CCN3 may mediate cell cycle arrest by bringing about senescence which is advantageous for the differentiation from CTB to STB and EVT. Furthermore, CCN3 may enhance invasion and migration capacities of the trophoblast by activating FAK/Akt pathway. Although an increase in p53 may increase the apoptosis of trophoblast cells, increased p21 may also promote cell viability through an anti-apoptotic pathway, which may contribute to the AIP disease. CCT, cell column trophoblast; EVT, extravillous trophoblast; STB, syncytiotrophoblast.

The schematic overview in **Figure 9** summarizes the proposed CCN3-mediated signaling pathways in placental diseases- early-onset PE and late after AIP-, based on the observed findings. The fusion from CTB to STB induces cellular senescence. EVT cells after leaving from the compact cell column have to escape from the cell cycle to stop proliferation, while invading into the maternal compartment. The precisely coordinated switch between senescence and invasion processes is needed for successful placental development. In the STB of early-onset PE, decreased CCN3 may decrease trophoblast migration ability and promote cell cycle progression by inhibiting FAK/Akt pathway and Notch-1/p21 signaling, thereby inhibiting cell fusion. In EVT cells of early-onset PE placentas, CCN3 immunofluorescence was barely detectable and seems to be involved in cell column trophoblasts (CCT) proliferation and their differentiation into EVTs. We speculate CCN3 may also enhance the invasion capacities of the EVT cells. Increased p53 expression can induce cell apoptosis and decreased p21 can also induce cell apoptosis which contribute to PE. However, in contrast, in late AIP placentas, CCN3 may mediate cell cycle arrest then bringing about senescence and enhancing invasion and migration capacities of the trophoblast by activating FAK/Akt pathway. Although an increase in p53 may increase the apoptosis of the trophoblast cells, increased p21 can also promote cell viability through an anti-apoptotic pathway, which may contribute to the AIP disease.

## Discussion

The matricellular CCN3 protein belongs to the CCN family, which has properties by binding to other molecules such as Notch-1 (44), Connexin43 (45), integrins and Fibulin 1C (46), and thus is involved in various biological processes. In our previous studies, we found that CCN3 seems to have multiple regulatory functions in trophoblast cells and triggers various placental developmental processes such as trophoblast differentiation, proliferation, migration and placental angiogenesis. All these cell biological issues are strictly controlled by CCN3 *via* the Akt and FAK signaling pathway as evidenced in trophoblast cell lines (31, 33).

This study demonstrated that under pathological pregnancy conditions such as in PE and AIP, CCN3 expression and the correlated signaling pathways are altered in placental tissues. CCN3 protein was co-expressed in the STB and EVT cells of controls, early preeclamptic placenta and AIP. Therefore, CCN3 may have a relationship with invasion properties of EVT cells as well as trophoblast differentiation processes in human placental development. In early-onset PE placentas, lower level of CCN3 protein was expressed compared to gestational age-matched controls, which is consistent with our previous report (47). The present study corroborates the fact that reduced CCN3 expression is associated with reduced invasion capacity of the EVTs. On the opposite, expression of higher levels of CCN3 in late AIP placentas points to the fact that CCN3 is involved in increased migration properties of the EVT. We have shown the mechanism in a former *in vitro* study in which we demonstrated that CCN3 could reduce cell proliferation through a G0/G1 cell cycle arrest and enhance the migration capability of human primary extravillous trophoblast SGHPL-5 cells by activating Akt and FAK kinases (33). Here, we explored that CCN3 altered the Akt and FAK kinases and Notch-1 signaling pathway by analyzing the expression of cleaved-Notch 1, pAkt, and pFAK and the expression of senescence markers probably associated with the development of PE or AIP. It was reported that the Akt-mTOR signaling pathway might contribute to the process of trophoblast cell fusion, but its mechanistic aspects still elude current knowledge (48). In our 2016 study (33), we have already shown that FAK and Akt enhanced migration capability in the trophoblast cell line SGHPL-5, and that CCN3 is able to activate FAK and Akt kinase pathway. Our immunofluorescence analysis lets assume that the CCN3 protein level is maybe lower in STB as well as EVTs of early-onset PE placentas compared to gestational-matched controls.

The mRNA and protein analyses in this study proved that CCN3 was lower in chorionic trophoblast tissue of early-onset PE placentas, which is consistent with our previous report (47). Here, we also found that activation of FAK-Akt-mTOR mediated signaling in early-onset preeclamptic placentas is significantly decreased, which may be one of the main factors affecting the differentiation and migration of trophoblastic cells in preeclampsia (19, 27).

It remains a challenge to prove if not only EVTs but also the STB contributes to the placental pathogenesis. The formation of STB requires the fusion of the underlying progenitor CTBs (49) and indicates the beginning of placental aging (21). Recent studies by Chuprin and colleagues (21) showed that the fusion from CTBs to STB induces cellular senescence, which may be necessary for proper STB function during embryonic development. Because senescent cells are resistant to apoptosis, this mechanism may maintain STB viability throughout pregnancy (50). Impairment of the fusion process leads to a large amount of STB apoptosis, necrotic material is released into the maternal blood combined with excessive maternal inflammatory reaction and transport of nutrients to the fetus is reduced (51, 52).

Senescence is in addition a critical process for the regulation of invasion because deeply invaded EVTs in the placental bed undergo senescence when having escaped from the cell cycle (53, 54) to prevent the development of choriocarcinoma. Thus, senescence plays an important regulatory role in normal pregnancy and dysregulation of the senescence pathways will lead to placental diseases, such as PE and AIP (22–24). Cellular senescence can be caused by multiple mechanisms (55–57) and is characterized by prolonged G1 cell cycle arrest. Several markers, including the cyclin-dependent kinase inhibitors (CDKIs) p21 and p16 (58) are senescence-related.

It is known from the literature that the developmental Notch pathway could be critically involved in human trophoblast differentiation (59–61). Notch is a cell-surface receptor, when binds to its ligand, causes the cleavage and release of the Notch intracellular domain (NICD), and enters the nucleus to regulate the transcription of Notch-regulated target genes (62). The Notch-1 receptor is expressed in cytotrophoblast cells of the human placenta (63). Notch signaling may be especially involved in controlling cell column trophoblasts (CCT) proliferation and their differentiation into EVTs (61). Sahin et al. (64) showed that in FGR and pregnancy-induced hypertension (PIH) placentas, the immunoreactivity of Notch-1 protein was significantly decreased in the brush border of cells of the STB layer, which suggested that Notch proteins may be associated with trophoblast differentiation (64). Our *in vitro* studies revealed that CCN3 up-regulates the expression of cleaved Notch-1/p21, allowing trophoblasts to escape from the cell cycle, resulting in enhanced migration and invasion ability (31, 33). Here, we found in early PE placentas that both the expression of CCN3 and cleaved Notch-1 is decreased. Therefore, it is possible that reduced CCN3 leads to decreased expression of cleaved Notch-1. Several studies report a decreased expression of Notch-1 in preeclamptic placentas (65–67), which is also significantly down-regulated in FGR combined with a reduction in placental weight (64).

Considered all these studies cleaved Notch-1 proteins might regulate STB and EVTs differentiation which was supported by our findings. In addition, we revealed that p53 was significantly elevated in early-onset PE placentas. This observation confirms other reports of increased p53 in PE and FGR complications (43, 65, 68, 69). Since p53 is involved in regulating apoptotic processes (70), increased p53 may lead to increased apoptosis of trophoblast cells (43). In a previous *in vitro* study, we showed that p21 acts as a target of cleaved Notch-1 and is integrated in the CCN3 signaling pathway (33). Here, in early PE, the senescence marker p21 but not p16 was down-regulated in placental tissues. Cobellis et al. (65) also reported that preeclamptic placentas revealed a down-regulation of Notch-1 combined with a decreased p21Cip1 expression in the STB (65). This simultaneous up-regulation of p53 and down-regulation of p21 appear unusual but an increased p21 expression can protect the cells from apoptosis by binding directly to procaspase-3 to inhibit apoptosis (71), or by repressing the activation of caspase-9 mediated by CDK in the mitochondria (72). Thus, the down-regulation of p21 is required to induce apoptosis. Similarly, a further *in vitro* study confirmed that p21 is down-regulated and p53 is up-regulated in trophoblast cells of preeclamptic placentas (68). In contrast to our findings, Cindrova-Davies et al. (73) found that p21 was not significantly different between preterm labor and early-onset PE placentas. This discrepancy can be explained by the study cohort. Most of our placental samples are combined with complications of FGR while in the study of Cindrova-Davies et al. (73), placental tissues are only exclusively taken from PE disease without FGR. The non-regulation of p16 in early PE within this context has also been described here (73).

However, it is important to note that the observed changes in cell cycle-regulated genes could also be influenced by the fact that most of the early-onset PE cases suffered from FGR in this cohort and thus could be due to both PE and FGR. It might be a limitation of our study that we did not separate the placentas in PE with and without FGR due to the low number of cases. We are also aware that in early-onset preeclampsia, in general, the placental tissues are influenced by stress responses during development which may affect the expression of cell cycle-regulated genes analyzed in this study. However, we confirmed in previous studies, as shown above, that CCN3 itself regulated these cell cycle-regulating pathways also in cell culture experiments of trophoblast cell lines (31, 33).

The cell cycle regulators Cyclin E1 and pRb were significantly elevated in early-onset PE placentas in villous CTB and STB. Ray and co-workers showed similar results, with an increase in Cyclin E1 expression levels in the CTB in early onset PE placentas leading to enhanced proliferation by aiding the G1 to S transition (25). In the early and middle G1 phase, cyclin D-CDK4/6 can induce Rb hypophosphorylation. In the late G1 phase, cyclin E-CDK2 further phosphorylates Rb into a hyperphosphorylated state, and Rb completely releases the E2F transcription factor promoting cells to enter the S phase (74). Therefore, we suggest that Cyclin E1 is able to phosphorylate pRb, and impede the fusion process from CTBs to STB. Thus, in the early-onset PE group, CCN3 expression is decreased, associated with decreased p21 and increased p53, Cyclin E1, and pRb levels, which may have a relationship with cellular senescence and apoptosis and are associated with the fusion from CTBs to STB.

In accordance with our previous study, the present study revealed no significant difference of CCN3, p16, p21, Cyclin D1, p53 and cleaved Notch-1 expression in late-onset PE compared to term placenta control tissues. However, some studies report telomere shortening and increased senescence markers in late PE, as well as in FGR compared to the term labor group (75, 76). The difference could be explained by using the placentas of different gestational ages and methods. Davy and co-workers used tissues of FGR and controls which gestational age varied from 38 to 40 weeks (75). In Londero et al. (76), the mean gestational age of late PE was (37.13 ± 1.93) and late controls were 34–42 weeks, while in our study, the gestational age of late-onset PE were 34.9–36.5 weeks and late controls were 36.5–40 weeks. Londero et al. (76) performed immunohistochemistry instead of detecting gene expression by qRT-PCR and western blot which may also contribute to this difference. In addition, the difference between the early and late-onset groups may be due to the different pathophysiology. Early-onset preeclampsia has been primarily associated with poor placentation and fetal growth restriction, whereas late-onset preeclampsia has been linked to maternal factors that cause disparities between maternal perfusion and placental/fetal metabolic demand and is mostly not associated with FGR (24, 77–79). Therefore, it is highly relevant to match the observed results with gestational age as a confounding factor—as we did—since various studies showed that gene regulation in placentas and trophoblasts are differently expressed dependent on gestational age. Also, fetal sex of the placenta could be a confounding factor, since the placentas from PE and AIP cases are of unequal amounts of fetal sexes compared to the corresponding controls (see **Table 1**). However, the comparison of CCN3 protein expression in male and female placentas did not show any differences (**Supplementary Figure S3**).

Our findings gained from immunofluorescence let us assume that CCN3 protein level was higher in both STB and EVTs in late AIP placental tissues (gestational age week at delivery > 34 weeks), which was consistent with qPCR and western blot results. Therefore, it is possible that the increase in CCN3 in AIP tissues may contribute to trophoblast differentiation processes as well as invasion properties of EVT cells. We postulated that the increased CCN3 is associated with increased expression of the senescence markers and the activation of Akt-mediated signaling, mTOR and FAK kinases. In late AIP placentas, both CCN3 and cleaved Notch-1 increased which is contrary to the results in early preeclamptic tissues. In addition, the results from immunofluorescence suggested that senescence markers p21 and p16 were higher expressed in nuclei in STB in late AIP compared to control placentas. The immunoblot data showed that the cell cycle regulators p53, p21, as well as p16 levels were significantly increased in late AIP placentas. From studies on tumor cell lines where overexpression of p53 is associated with Notch-1 activation and enhance tumor invasion (80), we assume that in trophoblast cells, the increased cleaved Notch-1 and p53 may trigger the invasiveness of trophoblast cells similarly. In late AIP, the simultaneous up-regulation of p21 and Cyclin D1 initially appears unusual in connection with a cell cycle arrest, however, decreased proliferation rate may not relate to a decreased expression of Cyclin D1 (81). The simultaneous up-regulation of p21 and Cyclin D1 has been shown also in senescent cells (82, 83). Consistent with our study Li et al. (26) reported that Cyclin D1 was up-regulated in *Placenta accreta*. The result that pRb was significantly decreased in late AIP placentas compared to late gestational age-matched controls could contribute to cellular senescence in late AIP. A recent study of Tzadikevitch Geffen et al. (22) corroborate our results in respect of a higher expression of senescence marker p21 in late *Placenta percreta* (mean gestational age week >34 weeks), but they measured a decrease in p16 and p53 expression. These discrepancies could be due to AIP patient cohorts because it remains a challenge to utilize a standardized tissue collection from different pathological stages of AIP with different villi invasion depth (*accreta*, *increta*, *and percreta*). A further limitation of this study is the low amount of AIP tissue samples available due to these rare clinical cases, however the number is quite similar to other studies (22).

Our results suggest that an increase in CCN3 leads to an increase in the activation of the Akt-mTOR kinase pathway in late AIP placentas. Thus enhanced activation of the FAK-Akt-mTOR kinase pathway in the late AIP placentas may affect the differentiation of CTB cells and increase the migration of trophoblast. It may also increase cell invasiveness which causes over invasion into the uterine wall. In addition, increased CCN3 binds to Notch-1 and thereby alters the expression of p53 and senescence markers which promote fusion from CTBs to STB. This process may be specifically involved in controlling cell column trophoblast (CCT) proliferation and their differentiation into EVT cells which need further study. In early AIP, the expression of CCN3 and senescence markers are only elevated by trend, leading to the suggestion that the early AIP pathology may be not as severe as the late form of deep trophoblast invasion observed in the late AIP subtype. Because in the third trimester of pregnancy, especially after 32–34 weeks, due to the combined effect of the fetal presentation, the lower segment of the uterus will be further elongated, and the myometrium will become thinner, especially in late *Placenta percreta* tissue (13).

In conclusion, we found that CCN3 was conversely expressed in early preeclampsia and late AIP placentas. As shown in **Figure 9**, the proposed molecular mechanisms of CCN3-mediated signaling pathway were related to the process of trophoblast proliferation, senescence and maybe also related to the EVT invasion properties. CTB fusion into STB for transplacental transport properties and CCT escape from cell cycle and differentiation into EVTs for invading into the maternal compartment and vessels are essential for the appropriate development of the placenta to establish a nutrition route to the fetus. These processes are closely associated with senescence, migration, and invasion abilities of the trophoblast cells. In early-onset PE, we assume that the deregulated expression of CCN3 may lead to an imbalance in the differentiation of STB and EVT through an altered FAK-Akt-mTOR and cleaved Notch-1/p21 signaling pathway. Moreover, down-regulation of CCN3 could prevent the conversion from CTB to STB by reducing senescence-related factors responsible for slowing down the senescence process of trophoblast cells concomitantly with an increase in p53 to promote apoptosis. Whereupon in late AIP disorders, the up-regulation of CCN3 may mediate cell cycle arrest resulting in senescence concomitantly by activating the FAK-Akt-mTOR pathway and the cleaved-Notch-1/p21, both may contribute to increasing EVT invasion properties. Thus, changes in CCN3 levels alter the expression of senescence markers and the FAK-Akt-mTOR pathway in placental disorders with reduced or increased the proliferation and/or differentiation of CTB. Whether it is possible to adjust the invasion properties in detail need further exploration. Thus, we provide here possible mechanisms to understand the reasons for the development of PE and AIP diseases.

## Data Availability Statement

The original contributions presented in the study are included in the article/**Supplementary Material**. Further inquiries can be directed to the corresponding author.

## Ethics Statement

The studies involving human participants were reviewed and approved by No.: 12-5212-BO, Ethical commission, University of Duisburg-Essen. The patients/participants provided their written informed consent to participate in this study. Written informed consent was obtained from the individual(s) for the publication of any potentially identifiable images or data included in this article.

## Author Contributions

AG, AK, EW, LD, MF, and RK contributed to study design and interpretation of data, manuscript drafting, and critical discussion. AK processed the placenta tissues. AK, BR, LD, and YW performed the experiments, analyzed the data, and prepared the ﬁgures and tables. All authors contributed to the article and approved the submitted version.

## Funding

The research of this study was supported by grants from the German Research Foundation (DFG: Wi 774/22-2 and GE-2223/2-1). We also are grateful to the China Scholarship Council (CSC) for giving funding to support the MD study of LD. We acknowledge support by the Open Access Publication Fund of the University of Duisburg-Essen.

## Conflict of Interest

The authors declare that the research was conducted in the absence of any commercial or financial relationships that could be construed as a potential conflict of interest.
