# Supplementary material for: CCN3 Signaling Is Differently Regulated in Placental Diseases Preeclampsia and Abnormally Invasive Placenta
Source: Front Endocrinol (Lausanne). 2020 Nov 16;11:597549. doi: 10.3389/fendo.2020.597549 (PMC7701218; doi:10.3389/fendo.2020.597549)
Supplement: Supplementary file 1 [file DataSheet_1.docx]

Supplementary Material

# Supplementary Tables 1 -3 (sTable 1-3)

Supplementary table 1 shows patient characteristics of AIP cases with FIGO classification. Supplementary table 2 shows the pregnancy course of the early control group.

Supplementary table 3 shows the comparison of mRNA and protein data of CCN3, p16, p21 and Cyclin D1 expression in placentas of early- and late-onset preeclampsia and early and late AIP.

# Supplementary Figures 1-3

**Supplementary Figure 1.** **Protein expression of pRb, Cyclin E1, pFAK, pAkt and pmTOR in early PE placentas**

**Fig. S1 (A)** Representative western blot of protein expression in the early control group (N=7) compared to the early PE group (N=16). **(B-F)** Cyclin E1 and pRb protein levels as relative levels compared to Actin; pAkt, pFAK, pmTOR levels normalized to Akt, FAK, mTOR expression respectively and then normalized to a same sample run on each gel. Cyclin E1 and pRb protein levels was significantly increased while the pAkt/Akt, pFAK/FAK and pmTOR/mTOR were significantly decreased in early PE group. Data represent means ± SD. *p < 0.05 significantly up-/down-regulated compared to the control. +p > 0.05 indicates that there is no significant difference between the groups.

**Supplementary Figure 2. Protein expression of pRb, Cyclin E1, pFAK, pAkt and pmTOR in late AIP placentas**

**Fig. S2 (A)** Representative western blot results of protein expression in the late control group (N=10) and late AIP group (N=4). ac, in and per represent *Placenta accreta*, *Placenta increta* and *Placenta percreta* samples. **(B-F)** Cyclin E1 and pRb levels as relative levels compared to Actin; pAkt, pFAK, pmTOR levels normalized to Akt, FAK, mTOR expression respectively and then normalized to a same sample run on each gel. pRb protein levels were significantly decreased while pAkt/Akt, pFAK/FAK and pmTOR/mTOR were significantly increased in late AIP group. Cyclin E1 was not significant different between the two groups. l-co: late control; ac: *Placenta accreta*; ic: *Placenta increta* and per: *Placenta percreta*. Data represent means ± SD. *p < 0.05 and **p < 0.01 significantly up-/down-regulated compared to the control. +p ≥ 0.05 indicates that there is no significant difference between the groups.

**Supplementary Figure 3:** **Protein expression of CCN3 in PE and AIP placentas separated in male and female placentas**

**Fig. S3 (A)** Representative western blot results of CCN3 protein expression in the early control (M) group (N=3) vs early PE (M) group (N=6) and early control (F) group (N=4) vs early PE (F) group (N=10). M = male, F = female. The expression of CCN3 was significantly lower in both early PE groups independent from fetal sex. No significant difference was shown in early control (M) vs early control (F) and early PE (M) vs early PE (F). **(B)** Representative western blot results of CCN3 protein expression in the late control (M) group (N=5) vs late PE (M) group (N=6) and late control (F) group (N=5) vs late PE (F) group (N=3). **(C)** Representative western blot results of CCN3 protein expression in the early control (M) group (N=3) vs early AIP (M) group (N=2) and early control (F) group (N=4) vs early AIP (F) group (N=2). There were no significant differences among these groups. **(D)** Representative western blot results of CCN3 protein expression in the late control (M) group (N=5) vs late AIP (M) group (N=1) and early control (F) group (N=5) vs early AIP (F) group (N=3). There were no significant differences among these groups **(B-D)**. The expression of CCN3 was significantly increased in the late AIP (F) group. CCN3 protein in each placenta is expressed as a relative level compared to Actin and normalized to a same reference sample run on each gel. Data represent means ± SD. *p < 0.05 and **p < 0.01 significantly up-/down-regulated compared to the control. +p ≥ 0.05 indicates that there is no significant difference between the groups.
