# Supplementary figures and images for: CCN3 Signaling Is Differently Regulated in Placental Diseases Preeclampsia and Abnormally Invasive Placenta
Source: Front Endocrinol (Lausanne). 2020 Nov 16;11:597549. doi: 10.3389/fendo.2020.597549 (PMC7701218; doi:10.3389/fendo.2020.597549)

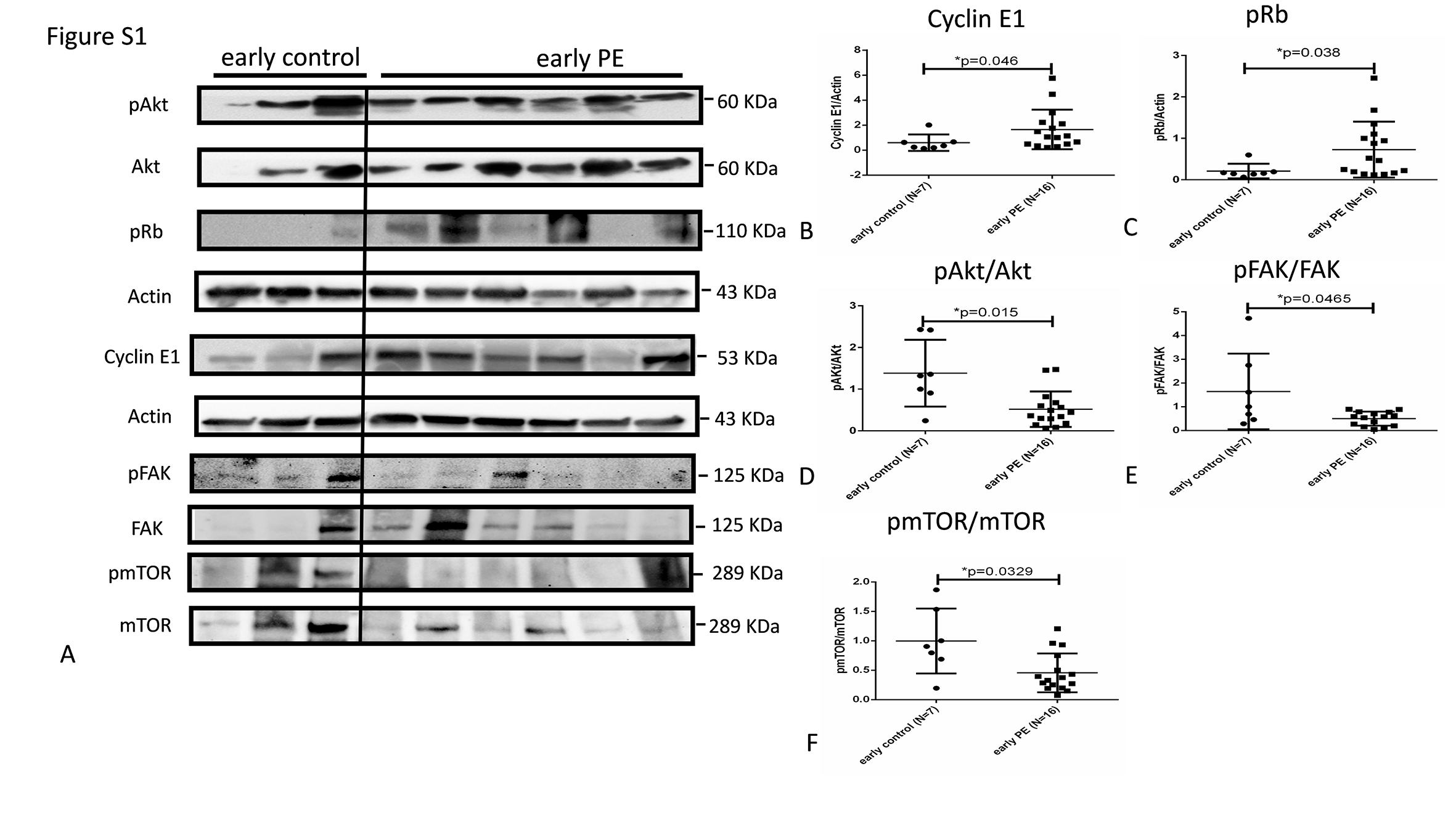

Supplement: Supplementary file 2 [file Image_1.tif]

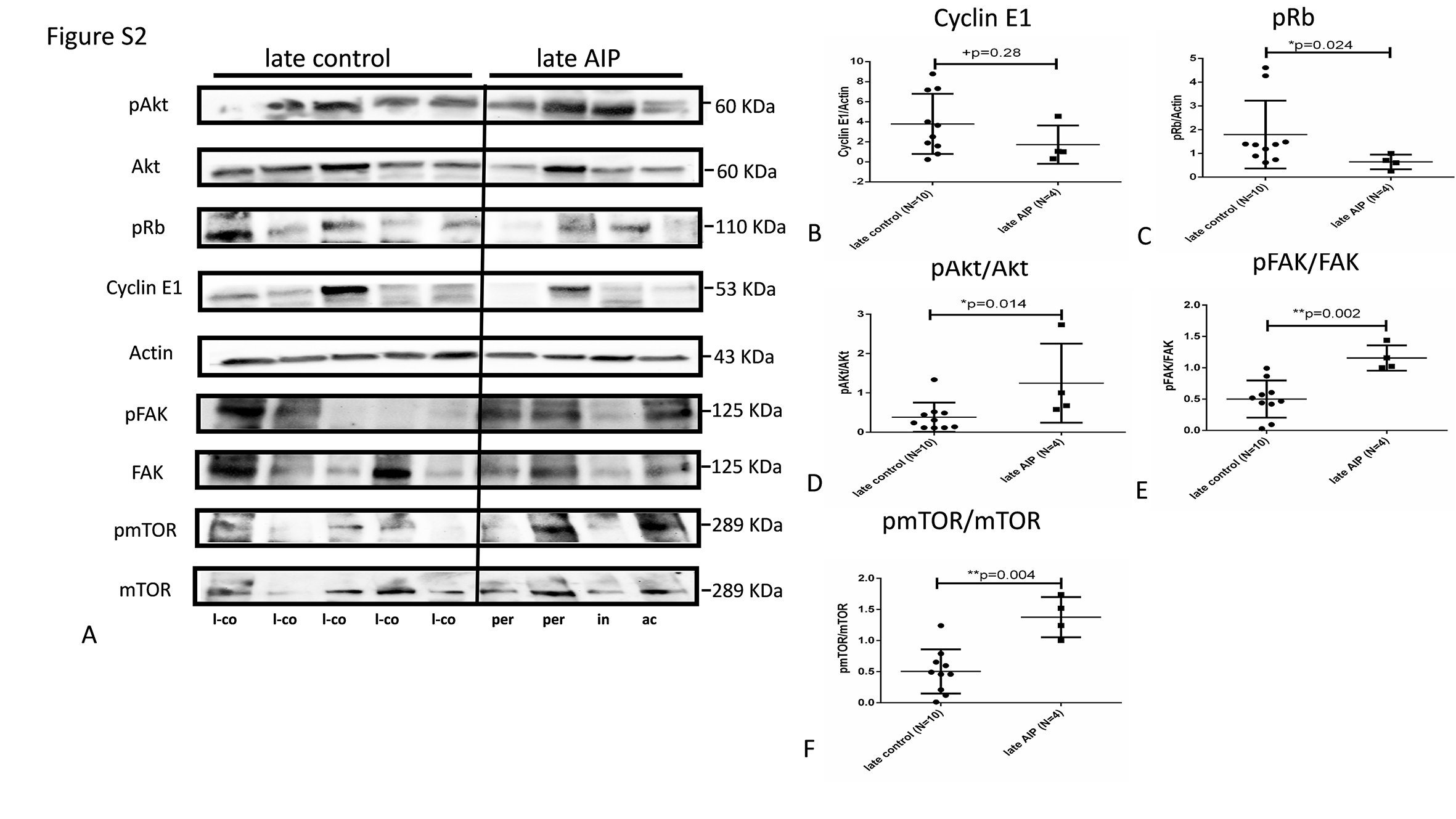

Supplement: Supplementary file 3 [file Image_2.tif]

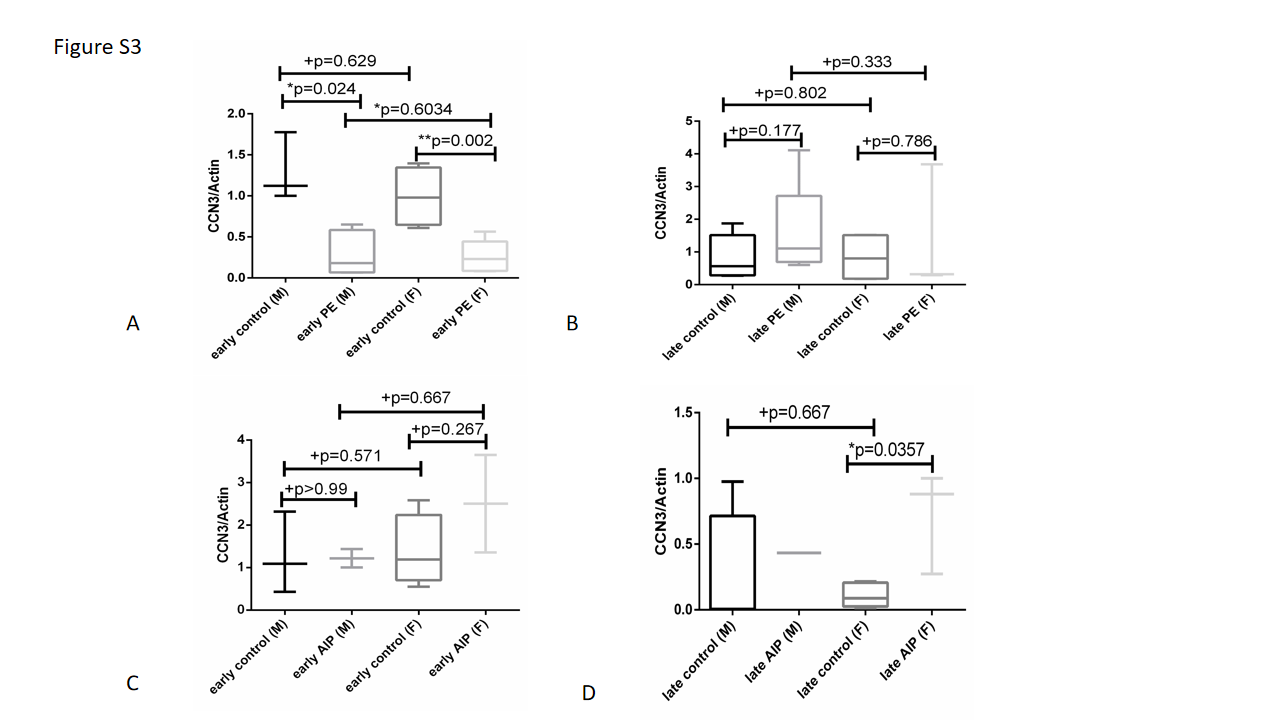

Supplement: Supplementary file 4 [file Image_3.tif]
