# Supplementary material for: CCN3 Signaling Is Differently Regulated in Placental Diseases Preeclampsia and Abnormally Invasive Placenta
Source: Front Endocrinol (Lausanne). 2020 Nov 16;11:597549. doi: 10.3389/fendo.2020.597549 (PMC7701218; doi:10.3389/fendo.2020.597549)
Supplement: Supplementary file 5 [file Table_1.docx]

**Supplementary Table 1 (sTable1): Pregnancy course of the AIP study cohort**

| **Patient number** | **Patient age (years)** | **Number of born children (including the AIP pregnancy)** | **Gestational age at birth (weeks and days post menstruation)** | **History of previous pregnancies and mode of delivery in the past** | **FIGO classification of AIP** | **Characteristics of the placenta position and mode of delivery** |
| --- | --- | --- | --- | --- | --- | --- |
| 1 | 37 | 2 | 38+0 | Post-partum curettage because of placenta accreta in the first pregnancy. | 1 *accreta* | Post-partum curettage. |
| 2 | 36 | 2 | 32+2 | Uneventful previous pregnancy with vaginal delivery. | 1 *accreta* | Post-partum curettage. |
| 3 | 36 | 6 | 39+5 | 5 CS | 2 *increta* | Focal excision of the increta-area. |
| 4 | 32 | 3 | 30+3  GA at tissue collection: 39+5 | 2 CS | 3c *percreta* | Ceasarean scar pregnancy with involution of the cervix uteri and the broad ligament.  Fundal incision for delivery of the baby at 30+3 weeks of pregnancy; LISA with emergency hysterectomy after 8 weeks due to uterine contractions with cervical dilatation. |
| 5 | 39 | 2 | 28+1 | Two spontaneous deliveries; 5 curettages due to *Placenta accreta* and two miscarriages. | 3a *percreta* | Dichorial twin pregnancy: one fetus with intrauterine demise because of IUGR; isthmo-cervical implantation of the second AGA-twin. Fundal incision for delivery of the babies, planned intra partum- hysterectomy. |
| 6 | 38 | 2 | 30+0 | 2 CS | 3c *percreta* | Ceasarean scar pregnancy with involution of the cervix uteri and the broad ligament.  Fundal incision for delivery of the baby, planned intra partum- hysterectomy. |
| 7 | 34 | 2 | 36+1 | 1 CS | 3b *percreta* | Ceasarean scar pregnancy with involvement of the bladder wall, placenta *Praevia totalis*.  Fundal incision for delivery of the baby and planned LISA, emergency intra partum- hysterectomy due to severe vaginal bleeding. |
| 8 | 41 | 2 | 36+0 | 1 CS | 3a *percreta* | Cesarean scar pregnancy. Fundal incision for delivery of the baby, emergency intra partum- hysterectomy due to severe bleeding of the lower uterine segment after focal resection of AIP. |

**Supplementary Table 2 (sTable2): Pregnancy course of the early control study cohort**

| **Patient number** | **Patient age (years)** | **Number of born children (including the current pregnancy)** | **Gestational age at birth (weeks and days post menstruation)** | **History of previous pregnancies and mode of delivery in the past** | **Characteristics of the current pregnancy and mode of delivery** |
| --- | --- | --- | --- | --- | --- |
| 1 | 32 | 2 | 32+6 | Uneventful previous pregnancy with vaginal delivery. | Placental abruption, emergency CS. |
| 2 | 33 | 3 | 30+2 | Two uneventful previous pregnancies with vaginal delivery. | Glioblastoma of mother, emergency CS because of life-limitating neurological complications. |
| 3 | 43 | 1 | 33+6 | No previous pregnancy. | *Placenta praevia* with bleeding. |
| 4 | 45 | 2 | 31+6 | IUGR pregnancy in the past. | CS due to severe IUGR. |
| 5 | 28 | 6 | 33+1 | 5 uneventful previous pregnancies with vaginal delivery | Placental abruption, emergency CS. |
| 6 | 30 | 1 | 25+5 | No previous pregnancy. | Emergency CS because of preterm labor with cervical dilatation, breech presentation and amnioninfection syndrome. |
| 7 | 24 | 2 | 32+2 | One CS only 4 weeks before conception of the current pregnancy. | CS because of imminent uterine rupture after quick succession of pregnancies. |

Abbreviations: AIP= abnormal invasive placenta, CS= cesarean section, GA= gestational age, LISA= leaving the placenta in situ approach, IUGR= intrauterine growth retardation, AGA= appropriate for gestational age

**Supplementary Table S3 (sTable 3):** Comparison of mRNA and protein data of CCN3, p16, p21 and Cyclin D1 expression in placentas of early- and late-onset preeclampsia and early and late AIP

| **Method** | **mRNA**  **(qPCR)** | | | | **Protein**  **(Western blot)** | | | |
| --- | --- | --- | --- | --- | --- | --- | --- | --- |
| **group** | **early**  **PE** | **late**  **PE** | **early**  **AIP** | **late**  **AIP** | **early**  **PE** | **late**  **PE** | **early**  **AIP** | **late**  **AIP** |
| **CCN3** |  | **nd** | **nd** |  |  | **nd** | **nd** |  |
| **p16** | **nd** | **nd** | **nd** |  | **nd** | **nd** | **nd** |  |
| **p21** |  | **nd** | **nd** |  |  | **nd** | **nd** |  |
| **Cyclin D1** |  | **nd** | **nd** | **nd** |  | **nd** | **nd** |  |

**early/late PE, early/late AIP are compared to gestational-matched controls early/late control**

**orange font: discordant results of Cyclin D1 in late AIP**

**nd= no difference significant decrease significant increase**
